# The complete chloroplast genome of *Androsace mariae*

**DOI:** 10.1080/23802359.2020.1866463

**Published:** 2021-02-08

**Authors:** Yupeng Guo, Rui Ma, Jun Xie, Qiujuan Liu, Ye Zhu, Wenjing Yang, Lina Gao, Yanjun Ma

**Affiliations:** a Qinghai Provincial Key Laboratory of High value Utilization of Characteristic Economic Plants, College of Ecological Environment and Resources, Qinghai Nationalities University, Xining, Qinghai, P. R. China; b College of Forestry, Gansu Agricultural University, Lanzhou, Gansu, P. R. China; c Ningxia Forestry Institute, Yinchuan, Ningxia, P. R. China

**Keywords:** *Androsace mariae*, chloroplast genome, phylogenetic analysis

## Abstract

The complete chloroplast genome of *Androsace mariae* was sequenced and assembled. It is a circular form genome of 151,958 bp in length, which was separated into four distinct regions, a large single-copy (LSC) of 83,292 bp, a small single-copy region (SSC) of 16,744 bp, two inverted repeats (IR) of 25,961 bp. After annotation, a total of 133 genes were predicted, of which, 87 were encoded proteins, 8 rRNA, and 37 tRNA. The evolutionary history, inferred using the neighbour-joining method, indicates that *A. mariae* was grouped within Primulaceae, and comprised a clade with other three species in *Androsace*, *Androsace paxiana*, *Androsace laxa* and *Androsace bulleyana*, with 100% bootstrap value.

*Androsace mariae*, belonging to Primulaceae, is an alpine perennial herbaceous plant with little red flowers. It is always found on the Qinghai–Tibet Plateau and adjacent highlands, it can survive at altitudes of 1800–4000 m (Delectis Florae Reipublicae Popularis Sinicae Agendae Academiae Sinicae Edita. 1989). Except Flora Reipublicae Popularis Sinicae, we have not found any other literatures of this plant. In this study, we reported its complete chloroplast (cp) genome.

Samples from Qilian mountains (36°34′37″N, 101°48′27″E) in Qinghai province were collected. Voucher specimen (GAUF20200531AMAR001) was deposited in the Herbarium, College of Forestry, Gansu Agricultural University. A sample was used for cp genome sequencing, in which the total DNA was extracted from fresh leaves and paired-end library was constructed. The sequencing was performed on the Illumina NovaSeq platform (Nanjing Jisihuiyuan biotechnology Co. Ltd). A total of 29,947,190 raw reads with 150-bp paired-end length were obtained. The complete cp genome was assembled with the *de novo* assembler SPAdes (Bankevich et al. 2012). Gene annotation was performed via prodigal v2.6.3 (Doug et al. 2010), hmmer v3.1b2 (hmmer.org, There is not yet any appropriate citable published paper that describes the HMMER3 software suite) and aragorn v1.2.38 (Dean and Bjorn 2004).

The cp genome of *A. mariae* (GenBank accession no. MT732944) has a typical quadripartite form of 151,958 bp in length, and composed of a large single-copy region (LSC, 83,292 bp), a small single-copy region (SSC, 16,744 bp), and two inverted repeats (IR, 25,961 bp). GC content of the genome is 37.27%. A total of 133 genes were predicted on this cp genome, of which, 87 were encoded proteins, 8 rRNA, and 37 tRNA.

Phylogenetic analysis was performed based on complete cp genomes of *A. mariae* and other 28 related species in Primulaceae, three species in Gentianaceae as outgroup. The sequences were aligned using MAFFT (Katoh et al. 2002) and trimAl was employed to remove ambiguously aligned sites (Capella-Gutierrez et al. 2009). The evolutionary history was inferred using the neighbour-joining method in MEGA7.0 (Kumar et al. 2016). Bootstrap (BS) values were calculated from 1000 replicate analysis (Figure 1). As expected, *A. mariae* was grouped within Primulaceae, and comprised a clade with other three species in *Androsace*, *Androsace paxiana*, *Androsace laxa*, and *Androsace bulleyana*, with 100% BS value. The complete cp genome of *A. mariae* will be helpful for further studies on population genetics, taxonomy or resources protection.

**Figure 1.**
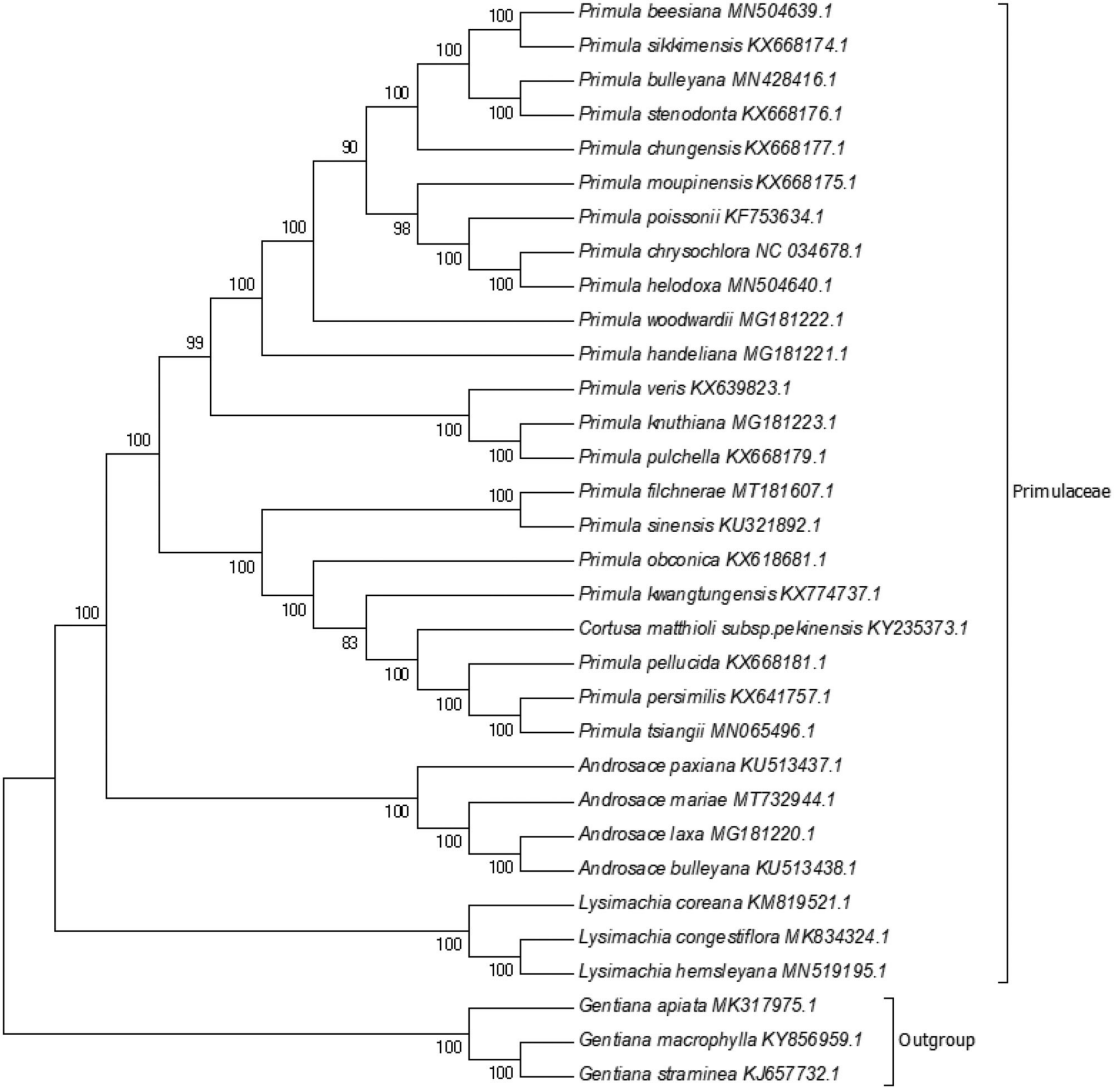
NJ phylogenetic tree based on 32 species chloroplast genomes was constructed using MEGA7.0. Numbers on each node are bootstrap from 1000 replicate.

## Data Availability

All Illumine reads supporting this research have been submitted to the NCBI Short Read Archive with BioProject ID: PRJNA678464 (https://www.ncbi.nlm.nih.gov/bioproject/PRJNA678464). The cp genome assembled and annotated is available in GenBank with accession number: MT732944 (https://www.ncbi.nlm.nih.gov/nuccore/MT732944.1).
